# Association between urate-lowering therapies and cognitive decline in community-dwelling older adults

**DOI:** 10.1038/s41598-022-17808-6

**Published:** 2022-09-12

**Authors:** Luc Molet-Benhamou, Kelly Virecoulon Giudici, Philipe de Souto Barreto, Christelle Cantet, Yves Rolland, MAPT/DSA group, Sandrine Andrieu, Christelle Cantet, Nicola Coley

**Affiliations:** 1 Gerontopole of Toulouse, Institute of Ageing, Toulouse University Hospital (CHU Toulouse), 31000 Toulouse, France; 2 CERPOP UMR1295, Université de Toulouse, Inserm, UPS, Toulouse, France; 3 CHU Toulouse, Toulouse, France; 4 CHU de Toulouse, Toulouse, France; 5 University of Seattle, Seattle, USA; 6 University of Montreal, Montreal, Canada; 7 CHU de Bordeaux, Bordeaux, France; 8 Hospital of Castres, Castres, France; 9 CHU de Dijon, Dijon, France; 10 Hospital of Foix, Foix, France; 11 Hospital of Lavaur, Lavaur, France; 12 University Hospital of Limoges, Limoges, France; 13 Centre Hospitalier Lyon-Sud, Lyon, France; 14 Hospital of Princess Grace, Monaco, France; 15 Hospital of Montauban, Montauban, France; 16 University Hospital of Montpellier, Montpellier, France; 17 Montpellier Univeristy Hospital, Montpellier, France; 18 University Côte d’Azur, Montpellier, France; 19 Hospital of Tarbes, Tarbes, France; 20 CATI Multicenter Neuroimaging Platform, Gif-sur-Yvette, France; 21 Bordeaux, France

**Keywords:** Neurology, Rheumatology, Risk factors

## Abstract

Long-term use of urate-lowering therapies (ULT) may reduce inflammaging and thus prevent cognitive decline during aging. This article examined the association between long-term use of ULT and cognitive decline among community-dwelling older adults with spontaneous memory complaints. We performed a secondary observational analysis using data of 1673 participants ≥ 70 years old from the Multidomain Alzheimer Preventive Trial (MAPT Study), a randomized controlled trial assessing the effect of a multidomain intervention, the administration of polyunsaturated fatty acids (PUFA), both, or placebo on cognitive decline. We compared cognitive decline during the 5-year follow-up between three groups according to ULT (i.e. allopurinol and febuxostat) use: participants treated with ULT during at least 75% of the study period (PT ≥ 75; n = 51), less than 75% (PT < 75; n = 31), and non-treated participants (PNT; n = 1591). Cognitive function (measured by a composite score) was assessed at baseline, 6 months and every year for 5 years. Linear mixed models were performed and results were adjusted for age, sex, body mass index (BMI), diagnosis of arterial hypertension or diabetes, baseline composite cognitive score, and MAPT intervention groups. After the 5-year follow-up, only non-treated participants presented a significant decline in the cognitive composite score (mean change − 0.173, 95%CI − 0.212 to − 0.135; *p* < 0.0001). However, there were no differences in change of the composite cognitive score between groups (adjusted between-group difference for PT ≥ 75 vs. PNT: 0.144, 95%CI − 0.075 to 0.363, *p* = 0.196; PT < 75 vs. PNT: 0.103, 95%CI − 0.148 to 0.353, *p* = 0.421). Use of ULT was not associated with reduced cognitive decline over a 5-year follow-up among community-dwelling older adults at risk of dementia.

## Introduction

Cognitive decline is a rising issue as the population of older adults keeps increasing. Inflammaging, defined as a chronic, sterile and low-grade inflammation^1^, can lead to age-related diseases. Neuroinflammation involving oxidative stress has been included in the inflammaging process and is reported to play a major role^2^ on cognitive impairment.

Though the hypothesis has been raised that hyperuricemia or gout may be protective against dementia, Alzheimer’s disease (AD) and cognitive decline^3,4^, results were mainly issued from cross-sectional studies and are somewhat conflicting^5^. It was also reported in a large cohort that gout was independently associated with a 15% higher risk of incident dementia among older adults^6^.

Publications have reported that urate-lowering therapies (ULT) may decrease the systemic inflammation and reduce the production of oxidative species^7,8^. ULT may also reverse endothelial dysfunction and thus have cardioprotective benefits. Neuroprotective effects of ULT both in animal studies^9^ and human cohorts^10^ have been reported. Singh et al*.*^11^ reported a dose-related reduction in the risk of dementia among people older than 65 years treated with ULT.

These studies remain scarce and controversial. Some of their limitations were the lack of evaluation criteria of cognitive decline^12–15^ and short-term follow-ups. Furthermore, analyses were based on general data of the health administrative database. In addition, participants investigated in these studies were not at higher risk of cognitive decline or AD, thus limiting the possibility of studying the link between cognitive decline and ULT.

The aim of the present study was to investigate the association between long-term ULT and cognitive decline in a sample of community-dwelling older adults with subjective memory complaints over a five-year follow-up.

## Materials and methods

### Study population

To evaluate a potential association between ULT and cognitive decline, we used data from the Multidomain Alzheimer Preventive Trial (MAPT) Study. The design of MAPT has been previously described in details elsewhere^16^. Briefly, the MAPT study is a randomized clinical trial performed in 13 centers in France and Monaco assessing cognitive outcomes in patients at risk of cognitive decline, including subjects older than 70 years with the following criteria: spontaneous memory complaint, limitations in one Instrumental Activity of Daily Living (IADL) or slow gait speed (≤ 0.8 m/s). Participants were assigned to different interventions for 3 years, and then followed for additional 2 years. Interventions comprised supplementation with omega 3 polyunsaturated fatty acids, multidomain intervention (covering cognitive training, physical activity and nutrition counselling), or both. These three groups were compared to a placebo group. Follow-up visits were scheduled at 6 and 12 months and then every year to assess outcomes, diseases and corresponding treatments. Participants were invited to come with all their treatment orders.

In summary, no statistically significant difference has been noted in the evolution of the main primary outcome (a composite cognitive score) between the four groups at 3 years, after controlling for multiple comparisons^17^.

From the total of 1,679 participants originally analyzed in the MAPT Study, those with information on ULT were included in the present study (n = 1673).

### ULT data collection

The use of ULT (Anatomic Therapeutic Chemical code: M04AA01 for allopurinol or M04AA03 for febuxostat) were recorded at each visit. Following the 2016 EULAR recommendations for the management of gout^18^, allopurinol, a xanthine-oxidase (XO) inhibitor^19^, is the first line therapy for chronic gout and febuxostat (which is also a XO-inhibitor) should be used in second intention. Patients treated (PT) by uricosuric or uricolytic drugs or treated with colchicine were not included in the treated groups, given the different mechanism of action of these drugs^20^ (uricosuric drugs including benzbromarone, sulfinpyrazone, and probenecid block renal tubular urate reabsorption^21^ and are second-line therapies for gout).

Three different groups were defined according to the use of urate-lowering medication, as follows: participants treated with ULT during at least 75% (PT ≥ 75) of the study 5-year period (n = 51), participants treated with ULT for less than 75% (PT < 75) of the study (n = 31), and participants never treated (PNT) with ULT during the study (n = 1.591). Among those treated with ULT, 14 subjects had no treatment start date; we then considered that these subjects were treated since baseline or earlier.

### Outcome measure

Cognitive function was assessed by a composite cognitive score combining the mean Z-score of four tests exploring: (i) episodic memory (Free and Cued Selective Reminding test: sum of the free and total (free + cued) recalls), (ii) orientation (10 orientation items from the Mini Mental State Examination—MMSE test), and (iii) executive function (WAIS Digit Symbol Substitution Test, and Category Fluency Test).

Cognitive assessments were performed at inclusion, every six months during the first year and every year until the fifth year of follow-up.

### Confounding variables

To take into account potential confounding factors, the following variables were considered: age, sex, body mass index (BMI = weight (kg)/height (m)^2^), diagnosis of arterial hypertension or diabetes, physical activity (minutes per week) and allocation to MAPT intervention groups. History of smoking and alcohol intake was not included in the analysis because data were available for less than half of the participants^22^.

### Statistical analysis

To describe the population, means and standard deviation for quantitative variables, and frequencies and percentage for qualitative variables were used. To compare the characteristics at baseline between the three urate-lowering treatment groups (PT ≥ 75, PT < 75 and PNT), the Chi-square test or Fisher's exact tests (in the case of expected frequencies < 5) were used for the qualitative variables, and Fisher test or the non-parametric Kruskal–Wallis test were used for quantitative variables.

To study the change of the composite cognitive score over time according to the ULT groups, mixed linear models were performed to take into account the correlated structure of the data (intra-center and intra-subject correlation, with subjects nested into the center). We included as random effects a random center intercept, a random subject intercept, and a random subject slope.

This model was performed without adjustment with the following fixed effects: time, ULT groups and time × group interaction. An adjusted model was then performed by adding the above-mentioned confounding variables as fixed effects. The statistically significant difference at baseline in the composite cognitive score according to the treatment group was also considered in the mixed model. Time was treated as a continuous variable, with a cubic trajectory since the terms time^2^ and time^3^ were significant.

All analyses were performed using SAS software version 9.4 (SAS Institute Inc., Cary, NC), and results were considered significant if *p* < 0.05.

### Ethical approval and consent to participate

All participants were recruited by the investigating physicians, after obtaining their written informed consent. The trial protocol was approved by the French Ethical Committee located in Toulouse, France (CPP SOOM II) and was authorized by the French Health Authority in 2007. All methods were performed in accordance with the relevant guidelines and regulations.

### Consent for publication

The publication was approved by the MAPT/DSA group.

## Results

Among the 1673 participants of the study, 1591 were not treated with ULT (PNT, 95%), whereas 82 were treated with ULT, with the following distribution: 31 in the group PT < 75 (2%) and 51 in the group PT ≥ 75 (3%). Baseline characteristics of the groups are reported in Table 1. In summary, both PT < 75 and PT ≥ 75 groups presented higher BMI, were older, more often hypertensive and more frequently male than the PNT group. Diabetes was significantly more prevalent in the PT ≥ 75 group compared to the other groups. The composite cognitive score at baseline were statistically different among the groups (0.00, SD = 0.67 for PNT; 0.30, SD = 0.56 for PT < 75; − 0.12, SD = 0.76 for PT ≥ 75; *p* = 0.019). Other parameters such as the inclusion criteria, allocation to MAPT intervention group, level of education, physical activity or the presence of the apolipoprotein E4 (APOE4) mutation, were not differently distributed between the groups.

**Table 1:** Participants’ characteristics at baseline.

Parameter at baseline	Total population(N = 1673)		PNT (n = 1591)		PT < 75 (n = 31)		PT ≥ 75 (n = 51)		*p* value ‡
	N	Mean (SD) or percentage	N	Mean (SD) or percentage	N	Mean (SD) or percentage	N	Mean (SD) or percentage	
**Demographic data**									
Age (years)		75.33 (4.42)		75.26 (4.38)		77.13 (5.36)		76.43 (4.74)	***0.03***
Sex (women)	1085	64.85%	1062	66.75%	13	41.94%	10	19.61%	** < *0.0001***
Education level (N = 1638)*									0.68
No diploma or primary school certificate	369	22.53%	355	22.80%	4	12.90%	10	20.00%	
Secondary education	553	33.76%	528	33.91%	10	32.26%	15	30.00%	
High school diploma	242	14.77%	230	14.77%	4	12.90%	8	16.00%	
University level	474	28.94%	444	28.52%	13	41.94%	17	34.00%	
**Clinical data**									
Arterial hypertension	899	53.74%	830	52.17%	28	90.32%	41	80.39%	** < *0.0001***
Diabetes	160	9.56%	143	8.99%	2	6.45%	15	29.41%	***0.0001***
BMI (N = 1666)*		26.11 (4.08)		26.00 (4.06)		27.40 (3.97)		28.96 (3.48)	** < *0.0001***
Fried score > 0 (N = 1603)*	738	46.04%	694	45.45%	17	56.67%	27	58.70%	0.10
ApoE4 carriers (N = 1298)*	299	23.04%	286	23.18%	5	20.83%	8	20.00%	0.87
Composite cognitive score	1673	0.00 (0.67)	1591	0.00 (0.67)	31	0.30 (0.56)	51	-0.12 (0.76)	**0.0185**
Physical activity (minutes per week)	1651	422.50 (405.04)	1571	426.49 (407.56)	30	398.58 (396.46)	50	311.30 (309.46)	0.06
**Inclusion criteria**									
Gait speed (m/s)		1.09 (0.26)		1.09 (0.26)		1.09 (0.25)		1.12 (0.26)	0.63
Memory complaint	1659	99.16%	1578	99.18%	30	96.77%	51	100.00%	0.28
IADL < 8	184	11.00%	176	11.06%	2	6.45%	6	11.76%	0.78
**Imaging data**									
Florbétapir-PET > 0 (N = 270)*	103	38.15%	97	37.89%	3	42.86%	3	42.86%	1.00
**Intervention group**									0.68
Omega 3 supplementation + Multidomain intervention	414	24.75%	390	24.51%	9	29.03%	15	29.41%	
Omega 3 supplementation only	419	25.04%	399	25.08%	10	32.26%	10	19.61%	
Multidomain intervention + placebo	420	25.10%	400	25.14%	8	25.81%	12	23.53%	
Placebo only	420	25.10%	402	25.27%	4	12.90%	14	27.45%	

*Number of subjects with available data.

^‡^Statistical tests are comparing the three groups PNT, PT < 75 and PT ≥ 75.

ApoE4: apolipoprotein E4; BMI: body mass index; PNT:participants not treated; PT < 75 :participants treated less than 75% of the study follow-up; PT ≥ 75: participants treated more than 75% of the study follow-up; *PET* positron emission tomography, *SD* standard deviation.

Significative values are written in bold text.

**Figure 1:**
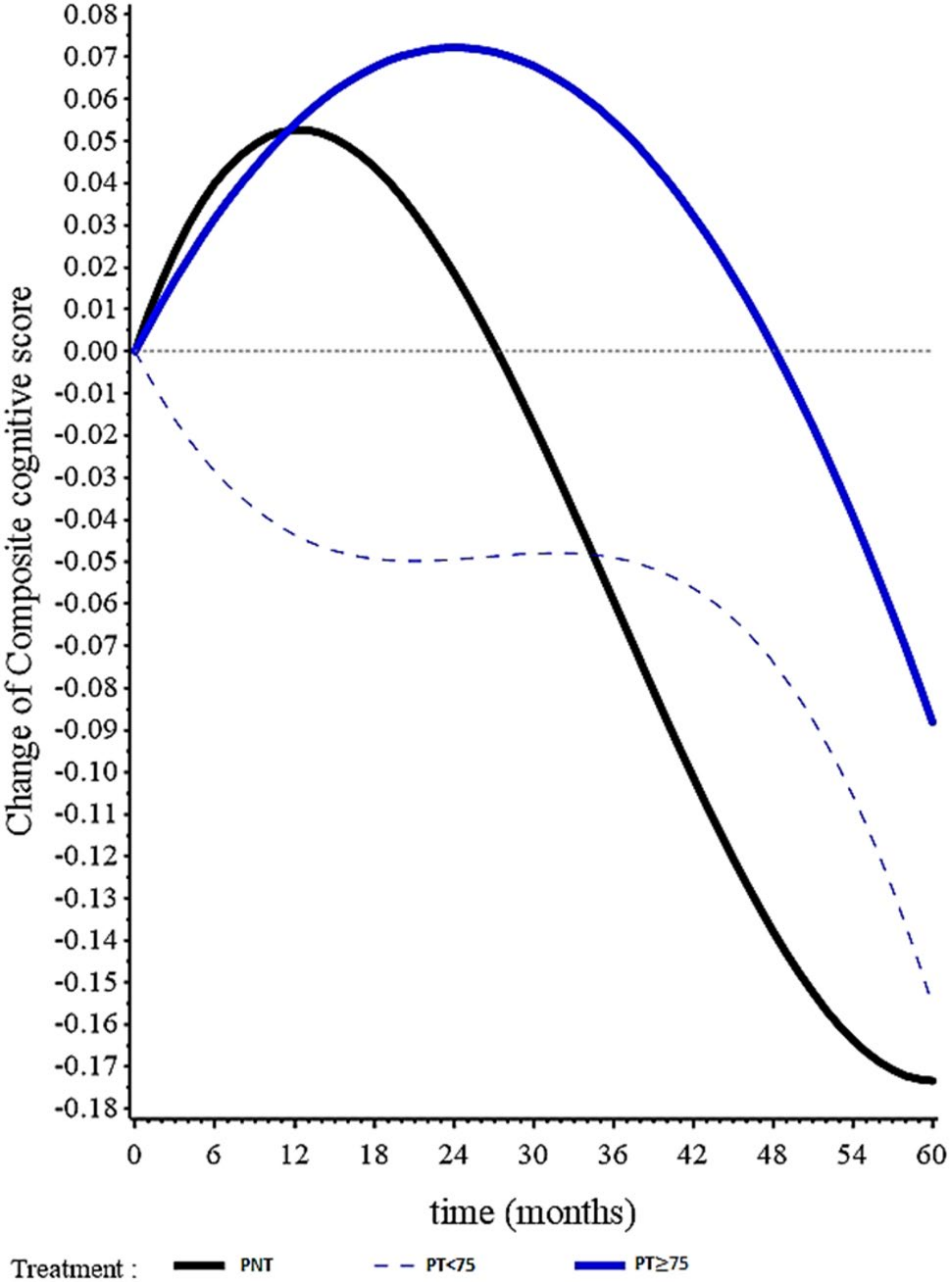
Evolution of the cognitive composite score (Z-Score) in the groups with participants not treated (PNT) with urate-lowering therapies (ULT), participants treated with ULT less than 75% of the study follow-up (PT < 75) and participants treated with ULT more than 75% of the study follow-up (PT ≥ 75) during a 5-year follow-up, among the participants of the MAPT study. PNT: patients not treated with urate-lowering therapies. PT < 75 : participants treated with urate-lowering therapies less than 75% of the study follow-up. PT ≥ 75 : participants treated with urate-lowering therapies more than 75% of the study follow-up.

After the 5-year follow-up, only the PNT group presented a significant decline in the composite cognitive score (mean change − 0.173, 95%CI − 0.212 to − 0.135, *p* < 0.0001) (Fig. 1 and Table 2).

**Table 2:** Linear mixed models presenting changes of the composite cognitive score according to urate-lowering therapies administration among community-dwelling older adults.

Time	Estimated change from baseline			Estimated differences in change from baseline		Estimated differences in change from baseline	
	PNT mean [95%CI] p-value	PT < 75% mean [95%CI] p-value	PT ≥ 75% mean [95%CI] p-value	PT < 75% vs PNT mean [95%CI] p-value	PT ≥ 75% vs PNT mean [95%CI] p-value	PT < 75% vs PNT mean [95%CI] p-value	PT ≥ 75% vs PNT mean [95%CI] p-value
	Not adjusted					Adjusted^(1)^	
3 years	− 0.060 [− 0.090 to − 0.029] *p* = 0.0002	− 0.049 [− 0.251 to 0.153] *p* = 0.633	0.055 [− 0.115 to 0.224] *p* = 0.528	0.010 [− 0.194 to 0.214] *p* = 0.920	0.114 [− 0.058 to 0.286] *p* = 0.194	0.061 [− 0.140 to 0.263] *p* = 0.551	0.162 [− 0.011 to 0.335] *p* = 0.066
5 years	− 0.173 [− 0.212 to − 0.135] *p* < 0.0001	− 0.155 [− 0.405 to 0.095] *p* = 0.223	− 0.088 [− 0.300 to 0.124] *p* = 0.414	0.018 [− 0.235 to 0.271] *p* = 0.889	0.085 [− 0.130 to 0.300] *p* = 0.437	0.103 [− 0.148 to 0.353] *p* = 0.421	0.144 [− 0.075 to 0.363] *p* = 0.196

PNT: participants not treated; PT < 75: participants treated less than 75% of the study follow-up; PT ≥ 75: participants treated more than 75% of the study follow-up.

^**(1)**^Adjustment for age, sex, body mass index (BMI), arterial hypertension, diabetes, allocation to MAPT intervention groups, baseline composite cognitive score and their interactions with time.

However, no differences were observed when comparing evolution of the composite cognitive score between the treated groups and the PNT group (PT ≥ 75 vs. PNT: 0.085, 95%CI − 0.130 to 0.300, *p* = 0.437; PT < 75 vs. PNT: 0.018, 95%CI − 0.235 to 0.271, *p* = 0.889). Results remained similar in the adjusted models (PT ≥ 75 vs. PNT: 0.144, 95%CI − 0.075 to 0.363, *p* = 0.196; PT < 75 vs. PNT: 0.103, 95%CI − 0.148 to 0.353, *p* = 0.421) (Table 2).

## Discussion

Our study evaluated the evolution of a composite cognitive score in a long follow-up of 5 years between participants treated with ULT and participants not treated with ULT, in a sample of community-dwelling older adults at risk of cognitive decline. To our knowledge, this is the first study assessing the long-term association of ULT with cognitive function in a population at risk of cognitive decline. No statistically significant differences in the evolution of a composite cognitive score was observed between the participants treated with ULT (whether they were treated during a long period of time or not) and the participants not treated with ULT.

Hyperuricemia is associated with several co-morbidities such as chronic kidney disease^23^, cardiovascular heart disease, arterial hypertension^24^, obesity and diabetes^25^. An important factor that may link hyperuricemia with these diseases is its deleterious effect on small vessels^26^. Despite having extracellular antioxidant properties, the urate acid induces endothelial dysfunction inside the cell^27^, and lowering uric acid concentrations has been reported to reduce cardiovascular events in patients with chronic kidney disease^28^. Our research are in line with a recent large systematic review and meta-analysis involving 16,000 participants, that found no significant association between serum urate levels and cognition^29^.

Both allopurinol and febuxostat can cross the blood–brain barrier (with a probability of 99% and 79%, respectively^30^, calculated with the chemical absorption, distribution, metabolism, excretion, and toxicity—ADMET features of these drugs^31^) and can directly interfere with intracerebral metabolism and neuronal cell^32^. Allopurinol was reported to reduce oxidative stress and proinflammatory molecules in the vessels^33^, thus reducing vascular damages in the brain. This neuroprotective effect has been reported to be related to an inhibition of the nitrosative stress and an attenuation of microglia infiltration and astrocytes reactivation in a mouse model of cortical microinfarction^9^. The potential benefits of the neuroprotective effect of allopurinol have been currently investigated in a phase III clinical trial in the context of hypoxic-ischemic brain injury in neonatology^10^. Cerebral microinfarcts are very frequent in patients with mild cognitive decline^34^, vascular dementia and AD^35^. Our research, however, did not show that ULT was associated with a protection of cognitive function (i.e., slower cognitive decline).

Our study has several strengths. Our selected population reported subjective memory complaints, a well-known condition associated with higher risk of cognitive decline^36^. Another strength of our study is the use of a composite cognitive score based on multiple cognitive tests to evaluate cognitive functions, which has been repeatedly validated^37^. Moreover, the measures of cognitive function have been repeated at several time-points, helping us follow the different cognitive trajectories more accurately. On the other hand, some limitations should be addressed. First, this is a post-hoc observational study using data from a RCT that was not designed to test the effects of ULT on cognition. Second, the small number of subjects in the PT < 75 and PT ≥ 75 groups may have limited the power of the statistical analysis. Third, serum uric acid levels were not available, what impeded us of investigating their interaction with ULT. Fourth, dose–response associations could not be explored since ULT dosing was not available. Finally, although based in our clinical experience, the 75% cut-off for ULT taking was arbitrary; other cut-offs should be tested in future works.

It would be interesting to wonder what practical implications should be drawn if our study had shown significant results and ULT be considered a treatment to slowing cognitive decline. ULT require few clinical and biological controls, thus their use is considered easy to handle^38^, not to mention their advantageous cost-effectiveness^39^. Moreover, uricemia often stays above the recommended levels in gouty patients treated with ULT^40^. Pursuing a dose-escalation of this therapy could lead to dwindle oxidative stress, as Singh et al. pointed out a dose-related effect of ULT on the onset of dementia in their study^11^. Eventually, as dementia is a slow-developing process, longer durations of ULT administration (as seen in chronic gout treatment) should be necessary to observe effective changes in cognitive functions.

In conclusion, this study did not find significant associations between ULT and changes in cognitive function over time in a population of older adults at risk of cognitive decline. Given the small fraction of people under ULT, larger observational prospective studies are needed to examine the associations between ULT and cognition over time. Randomized controlled trials investigating the effects of ULT on the cognitive function of patients might shed light on this topic.

## Data Availability

Pr Yves Rolland and Pr Philipe Barreto, CERPOP, UMR1295, unité mixte INSERM—Université Toulouse III Paul Sabatier have the full access to the MAPT database. The datasets used and/or analysed during the current study available from the corresponding author on reasonable request.

## Abbreviations

AD: Alzheimer’s Disease
ADMET: Absorption, Distribution, Metabolism, Excretion, and Toxicity
APOE4: Apolipoprotein E4
BMI: Body Mass Index
IADL: Instrumental Activity of Daily Living
MAPT: Multidomain Alzheimer Preventive Trial
MMSE: Mini Mental Status Evaluation
PNT: Participants Not Treated
PT < 75: Participants Treated with ULT for less than 75% of the study
PT ≥ 75: Participants Treated with ULT during at least 75% of the study
SD: Standard Deviation
ULT: Urate-Lowering Therapies
